# A Stability Indicating HPLC Method for the Determination of Fluvoxamine in Pharmaceutical Dosage Forms

**Published:** 2015

**Authors:** Effat Souri, Hassan Donyayi, Reza Ahmad khaniha, Maliheh Barazandeh Tehrani

**Affiliations:** a Department of Medicinal Chemistry, School of Pharmacy and Drug Design and Development Research Center, Tehran University of Medical Sciences, Tehran, Iran.; b Department of Human Ecology, School of Public Health, Tehran University of Medical Sciences, Tehran, Iran.

**Keywords:** Fluvoxamine, HPLC, Stability indicating, Stress degradation

## Abstract

Fluvoxamine maleate is a selective serotonin reuptake inhibitor, which is used for the treatment of different types of depressive disorders. In the present study, a stability indicating HPLC method was developed and validated for the determination of fluvoxamine maleate. The chromatographic separation was carried out using a Nova-Pak CN column and a mixture of K_2_HPO_4_ 50 mM (pH 7.0) and acetonitrile (60: 40, v/v) as the mobile phase. Target compounds were detected using a UV detector set at 235 nm. The developed method was linear over the concentration range of 1-80 μg/ml with acceptable precision (CV values < 2.0%) and accuracy (error values < 1.6%). The degradation studies showed that fluvoxamine maleate is relatively unstable under acidic, basic and oxidative conditions and also when exposed to UV radiation. On the other hand, the bulk powder of fluvoxamine maleate was relatively stable when exposed to visible light or heat. The proposed method was successfully applied for the determination of active ingredient of fluvoxamine dosage form without any interference from tablet excipients.

## Introduction

**Figure 1:**
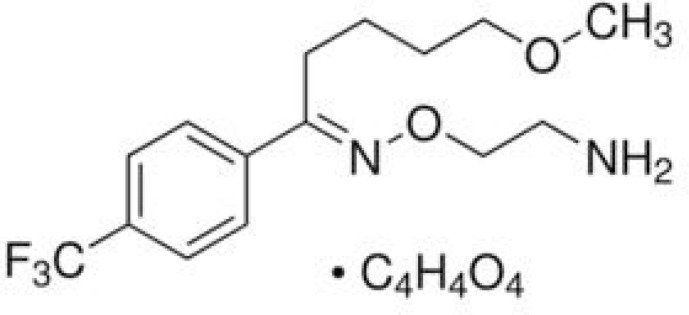
Chemical structure of fluvoxamine maleate

Fluvoxamine maleate, 5-methoxy-1-[4-(trifluoromethyl) phenyl]-1-pentanone-o-(2-aminoethyl) oxime maleate (Figure 1), which is effective in the treatment of different types of depression, increases the synapthic serotonin by selective inhibition of serotonin reuptake into presynaptic neurons (1).

Up to now several HPLC methods with UV detection have been developed for the determination of fluvoxamine in dosage forms (2-4). In some other reports spectro fluorimetric determination of fluvoxamine was performed in dosage Forms after derivatization with fluorescamine (5), or 7-chloro-7-nitrobezo-2-oxa-1, 3-diazole (6). Quantitative spectrophotometric (7) and polaro graphic methods (8) were also reported before for the analysis of fluvoxamine in dosage forms.

To the best of our knowledge, until now no report has been published regarding stress degradation of fluvoxamine maleate. The purpose of this study was to develop a simple and specific stability indicating HPLC method for the determination of fluvoxamine maleate in the presence of its degradation products. 

## Experimental

*Chemicals*

Fluvoxamine maleate was obtained from Sigma-Aldrich Chemie GmbH (Germany). HPLC grade solvents and other analytical grade chemicals were from Merck (Darmstadt, Germany). Fluvoxamine tablets (50 mg) were manufactured by Abidi Pharmaceutical Company (Tehran, Iran) and were purchased from a local pharmacy**.**

*Instrumentation*

The chromatographic analysis was carried out using a Waters HPLC system (Milford, USA) consisted of a Model 515 isocratic pump, a Model 710 plus auto sampler and a Model 480 UV-Vis detector. The data processing system was the version 1.5x of a multi-channel Chrom and Spec software for chromatography. A dry air oven (Melag, Germany) was used for thermal degradation studies. A 100 W Tungsten lamp and a low pressure Mercury lamp 100 W were used as visible or UV light source, respectively.

*Chromatographic conditions*

Separation was performed on a Nova-Pak CN column (150×3.9 mm, 4 μm, Waters, Milford, USA). A mixture of acetonitrile and 50 mM K_2_HPO_4_ (pH 7.0) (40:60, v/v) was used as the mobile phase. The flow rate was adjusted at 1 mL/min and peak detection was performed at 235 nm. The mobile phase was prepared daily and degassed by passing through a 0.45 μm filter and sonication for 5 min. 

A stock standard solution of fluvoxamine maleate (5000 μg/mL) was prepared in acetonitrile. Working standard solution (50 μg/mL) and calibration solutions (1, 2, 5, 10, 20, 40, 60 and 80 μg/mL) were prepared by subsequent dilution of the stock standard solution with mobile phase.

*Linearity*

Six series of standard calibration solutions of fluvoxamine maleate in the range of 1-80 μg/mL were prepared and analyzed by the developed method. The peak areas were plotted versus the concentrations of standard solutions. The linearity of the calibration curves was evaluated by linear regression analysis and the statistical data were calculated. 

The limit of quantification (LOQ) and limit of detection (LOD) were estimated using the following equations (9).

LOQ = 10/s and LOD = 3.3/s 

where   is the standard deviation of intercept and s is the slope of the calibration curve.

*Precision and accuracy*

The within-day and between-day precision and accuracy of the method were evaluated by analyzing three quality control samples at three different concentrations (2, 20 and 80 μg/mL) in triplicate in one day and three consecutive days, respectively. The concentration was determined using the calibration curve and the percentage of coefficient of variations (CV%) and error values were calculated.

*Robustness*

The effect of small variations in the pH value (± 0.3) and the amount of organic solvent of the mobile phase (± 5 mL) on the chromatographic conditions were studied to find out the robustness of the proposed method.

*Application of the proposed method*

An exact amount of the mixed finely powdered of 10 tablets, equivalent to one tablet, was transferred to a 100 mL volumetric flask. After addition of 70 mL of the mobile phase, the mixture was sonicated for 15 min and the flask completed to volume with the same solvent. A portion of the sample was centrifuged and filtered through a syringe filter (Teknokroma, Spain) and after dilution for ten times, 20 μL of the resulting solution was injected into the HPLC system in triplicate. The fluvoxamine maleate amount was calculated by comparison with a standard solution at the same concentration level.

*Recovery*

Assay samples were spiked by adding a known concentration of fluvoxamine maleate and analyzed. Control standard samples at the same concentration were prepared and the recovery was determined by comparison between these solutions.

*Stress degradation*

Stress degradation tests were performed according other reported articles (10, 11). The stability of fluvoxamine maleate was tested under different stress conditions including acid hydrolysis (0.5 M HCl at 80ºC for 10 min), basic hydrolysis (2 M NaOH at 80ºC for 40 min), and oxidation (10% H_2_O_2_ at 80ºC for 30 min). All degradation studies were performed at an initial concentration of 500 μg/mL in water containing 10% acetonitrile as co-solvent. The degradation samples were neutralized and diluted ten times with mobile phase and 20 μL of the resulted solution was injected into the HPLC system. The peak areas were compared with a reference standard solution of fluvoxamine maleate at the same concentration level.

To study the effect of light and heat, 100 mg of the bulk powder of fluvoxamine maleate were spread in a watch glass and exposed to visible and UV light and heat for 5 days. A working sample was prepared at the concentration level of 50 μg/mL and injected to the HPLC system. An aqueous solution of fluvoxamine maleate (500 μg/mL) using 10% acetonitrile as co-solvent was also exposed to light and heat. The percentage of degradation of all samples was calculated by using a standard solution of fluvoxamine maleate at the same concentration. 

## Results and Discussion

*Chromatographic conditions*

**Figure 2:**
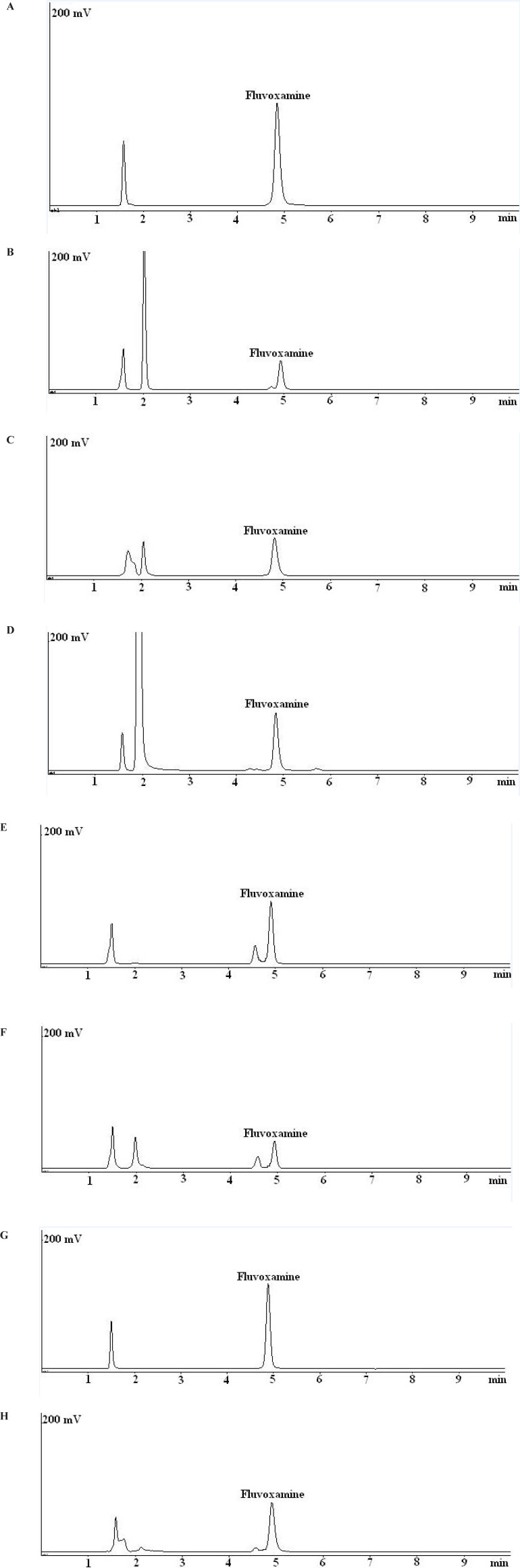
Typical chromatograms obtained from stability studies of fluvoxamine maleate. (a) fluvoxamine maleate standard solution (50 mg/mL); (b) fluvoxamine maleate solution in 0.5 M HCl after 10 min at 80 °C; (c) fluvoxamine maleate solution in 2 M NaOH after 40 min at at 80 °C; (d) fluvoxamine maleate solution in 10% H2O2 after 30 min at 80 °C; (e) fluvoxamine maleate bulk powder after 5 days exposure to UV light; (f) fluvoxamine maleate solution after 5 days exposure to UV light; (g) fluvoxamine maleate solution after 5 days exposure to visible light; (h) fluvoxamine maleate solution after 5 days exposure to heat

Unsatisfactory results were obtained using the C_18_ column and different composition of buffer and organic solvent as the mobile phase. On the other hand, by using a Nova-Pak CN column, sharp peaks and smooth baseline and acceptable separation were achieved. Representative chromatograms are shown in Figure 2. The system suitability parameters were calculated after six replicate injections of standard fluvoxamine maleate solution into the HPLC system. The results are shown in Table 1 which is in the acceptable range. 

**Table 1:** System suitability parameters

**Parameters**	**Found**	**Acceptable limits**
USP theoretical plates (n = 6)	5400	N>1500
USP tailing factor (n = 6)	1.11	T<1.5
Repeatability (t_R_) (n = 6)	0.89	RSD<1%
Repeatability (peak area)(n = 6)	0.65	RSD<1%

t_R_: Retention time (min); N: Theoretical plate; T: Tailing factor; RSD: Relative Standard Deviation

*Linearity*

The constructed calibration curves were linear over the concentration range of 1-80 μg/mL. The statistical data of replicate injections are shown in Table 2. The LOQ and LOD were found to be 1.28 and 0.42 μg/mL, respectively.

**Table 2:** Statistical data of calibration curves of fluvoxamine maleate (n = 6).

**Parameters**	**Results**
Linearity range	1-80 g/mL
Regression equation	y = 15.80 x-9.76
Standard deviation of slope	0.14
Relative standard deviation of slope (%)	0.87
Standard deviation of intercept	2.03
Correlation coefficient (r^2^)	0.9998
Limit of quantification (LOQ)	1.28 g/mL
Limit of detection (LOD)	0.42 g/mL

*Robustness*

Minor changes in the mobile phase composition and pH of the buffer did not produce major changes in the peak area or peak shape of fluvoxamine maleate or degradation products which indicates the robustness of the developed method.

*Precision and accuracy*

The within-day and between-day accuracy and precision of the method were evaluated by carrying out three independent determinations of fluvoxamine maleate solutions at three different concentrations in the calibration range in one day and three consecutive days. The results are shown in Table 3 which indicates high precision and accuracy of the proposed method.

**Table 3:** Precision and accuracy of the method for determination of fluvoxamine maleate (3 sets for 3 days).

**Concentration added ** **(g/mL)**	**Concentration found (g/mL)**	**CV (%)**	**Error (%)**
**Within day (n = 3)**			
2.00 20.00 80.00	2.03±0.04 19.73±0.14 79.09±0.68	1.97 0.70 0.87	1.55 -1.35 -1.14
**Between day (n = 9)**			
2.00 20.00 80.00	2.01±0.04 20.07±0.28 79.75±0.85	1.81 1.39 1.06	0.60 0.34 -0.31

*Application*

The proposed HPLC method was used for the determination of fluvoxamine maleate in tablet dosage form. The assay results showed 50.21 ± 0.59 mg of fluvoxamine maleate in one tablet which is in great accordance with the labeled amount (50 mg). No significant interfering peaks were observed from the tablet excipients which indicate method selectivity.

*Recovery*

The standard addition technique was used to find out the relative recovery of fluvoxamine maleate. The recovery was calculated by comparing the drug concentrations which were determined for spiked tablet samples with those of a standard solution. Results showed a mean percentage recovery of 98.77 ± 0.01%.

*Stress degradation*

Forced degradation studies were conducted on fluvoxamine maleate bulk powder. The degradation samples were injected into the HPLC system and the results are shown in Table 4.

**Table 4:** The results of the stress degradation tests on fluvoxamine maleate bulk powder using different conditions

**Stress test condition**	**Solvent**	**Temperature**	**Time**	**% of fluvoxamine**
**Acidic **	0.5 M HCl	80ºC	10 min	37.5
**Basic**	2 M NaOH	80ºC	40 min	55.4
**Oxidative **	10% H_2_O_2_	80ºC	30 min	74.3
**Photolytic**				
UV light UV light Visible light Visible light	Solid form Water Solid form Water	Room temperature Room temperature Room temperature Room temperature	5 days 5 days 5 days 5 days	73.8 27.1 92.9 82.4
**Heat **	Solid form Water	80 C 80 C	5 days 5 days	98.4 76.0

Fluvoxamine maleate was very susceptible to degradation under exposure to 0.5 M HCl at 80ºC and about 62% degradation was observed after 10 min. Under this condition, an unknown peak at the retention time of about 2.1 min was formed (Figure 2b). The rate of hydrolysis in basic conditions was slower than acidic conditions. About 45% degradation was observed under exposure to 2 M NaOH at 80ºC after 40 min. Apparently, the degradation product under basic or acidic conditions were identical (Figure 2c).

Fluvoxamine maleate was degraded under 10% H_2_O_2 _(about 26%) after 30 min and few minor degradation products were detected in the chromatogram (Figure 2d).

No significant degradation was observed when fluvoxamine maleate bulk powder exposed to heat for 5 days. Slight degradation (about 7%) was observed upon exposure of the bulk powder to visible light. On the other hand, fluvoxamine maleate bulk powder and its solution were sensitive to UV light. A major degradation product at the retention time of about 4.5 was observed under exposure to UV light (Figures 2e and 2f). The degradation rate in the fluvoxamine maleate solution is higher than bulk powder. The fluvoxamine maleate solution showed about 18% degradation under visible light without any new peak (Figure 2g). Upon exposure to heat, fluvoxamine maleate solution degraded about 24% and a new small peak observed before the drug peak (Figure 2h).
